# The protein tyrosine kinases EpsB and PtkA differentially affect biofilm formation in *Bacillus subtilis*

**DOI:** 10.1099/mic.0.074971-0

**Published:** 2014-04

**Authors:** Jan Gerwig, Taryn B. Kiley, Katrin Gunka, Nicola Stanley-Wall, Jörg Stülke

**Affiliations:** 1 Department of General Microbiology, Institute of Microbiology and Genetics, Georg-August University Göttingen, Grisebachstr. 8, D-37077 Göttingen, Germany; 2 Division of Molecular Microbiology, College of Life Sciences, University of Dundee, Dundee DD1 5EH, UK

## Abstract

The Gram-positive soil bacterium *Bacillus subtilis* is able to choose between motile and sessile lifestyles. The sessile way of life, also referred to as biofilm, depends on the formation of an extracellular polysaccharide matrix and some extracellular proteins. Moreover, a significant proportion of cells in a biofilm form spores. The first two genes of the 15-gene operon for extracellular polysaccharide synthesis, *epsA* and *epsB*, encode a putative transmembrane modulator protein and a putative protein tyrosine kinase, respectively, with similarity to the TkmA/PtkA modulator/kinase couple. Here we show that the putative kinase EpsB is required for the formation of structured biofilms. However, an *epsB* mutant is still able to form biofilms. As shown previously, a *ptkA* mutant is also partially defective in biofilm formation, but this defect is related to spore formation in the biofilm. The absence of both kinases resulted in a complete loss of biofilm formation. Thus, EpsB and PtkA fulfil complementary functions in biofilm formation. The activity of bacterial protein tyrosine kinases depends on their interaction with modulator proteins. Our results demonstrate the specific interaction between the putative kinase EpsB and its modulator protein EpsA and suggest that EpsB activity is stimulated by its modulator EpsA.

## Introduction

Like many other bacteria, the Gram-positive model organism *Bacillus subtilis* is able to form structured biofilms in order to attach to surfaces and to withstand harsh environmental conditions. Biofilm formation in *Bacillus subtilis* is usually observed as the formation of either structured complex colonies on solid surfaces or thick floating pellicles in liquid media (Branda *et al.*, 2001). *Bacillus subtilis* cells in the biofilm form an extracellular matrix composed of polysaccharides, amyloid-like fibres made up of the TasA protein, and a water-repellent surface coat formed by the bacterial hydrophobin BslA (see Vlamakis *et al.*, 2013 for a review). Interestingly, biofilm formation is strongly impaired in the domesticated laboratory strain of *Bacillus subtilis*, 168. This strain forms only poorly structured colonies and thinner and non-structured yet stable pellicles. Such a loss of phenotype, which is important in natural environments but not in the laboratory, is commonly observed in bacteria. Reduced biofilm formation in the *Bacillus subtilis* laboratory strain results from the presence of point mutations in the *sfp*, *swrAA*, *degQ* and *epsC* genes and from the absence of a plasmid that harbours the *rapP* gene encoding a response regulator aspartate phosphatase. Introduction of wild-type copies of these genes restores biofilm formation in the domesticated laboratory strain (McLoon *et al.*, 2011).

Expression of the *epsA-O* operon encoding the enzymes for extracellular polysaccharide synthesis and of the *tapA–sipW–tasA* operon encoding the amyloid-like protein and the factors for its export and assembly is under tight control by the RemA transcription activator and the SinR repressor protein (Chu *et al.*, 2006; Winkelman *et al.*, 2013). The DNA-binding and thus repressing activity of SinR is controlled by regulatory protein–protein interactions with one of three antagonist proteins: SinI, SlrA and SlrR (Chai *et al.*, 2009, 2010; Lewis *et al.*, 1998; Newman *et al.*, 2013). The interaction between SinR and SlrR seems to be of key importance as the complex not only prevents the repression of biofilm gene expression but also acts as a transcriptional repressor for motility and autolysis genes. This mechanism ensures that a single cell of *Bacillus subtilis* has to choose to express either biofilm or motility genes but not both sets (Chai *et al.*, 2010). The choice of cell fate is also affected by the recently discovered YmdB protein, which is essential for the expression of biofilm genes (Diethmaier *et al.*, 2011, 2014).

While the functions of the proteins of the *tapA–sipW–tasA* operon have been studied in detail, far less is known about the individual functions of the proteins encoded by the 15-gene *epsA-O* operon. For the EpsE protein, it was shown that it is required for extracellular polysaccharide synthesis, but that it also inhibits motility by separating the cytoplasmic FliG motor from the MotA–MotB stator (Blair *et al.*, 2008; Guttenplan *et al.*, 2010). The proteins encoded by the two promoter-proximal genes of the *eps* operon, EpsA and EpsB, are similar to bacterial protein tyrosine kinases (BY-kinases). Interestingly, these BY-kinases are present as single multidomain proteins in Gram-negative bacteria, whereas the kinase and its dedicated transmembrane modulator are separated proteins in Gram-positive bacteria (Grangeasse *et al.*, 2012).

*Bacillus subtilis* encodes two potential pairs of BY-kinases (PtkA and EpsB) and their modulators (TkmA and EpsA, respectively). While the activity of PtkA/TkmA has been intensively studied, nothing is known about the function(s) of EpsB and its modulator EpsA. For PtkA, it has been established that the kinase phosphorylates and thereby activates the UDP-glucose dehydrogenases Ugd and TuaD as well as the single-stranded DNA-binding proteins SsbA and SsbB (Mijakovic *et al.*, 2003; Petranovic *et al.*, 2007). In addition, PtkA-dependent phosphorylation decreases the DNA-binding activity of the transcription repressor FatR (Derouiche *et al.*, 2013). Moreover, PtkA-dependent protein phosphorylation was proposed to affect protein localization, as observed for the glycolytic enzyme enolase (Jers *et al.*, 2010). Interestingly, the BY-kinase PtkA is attached to its membrane modulator during vegetative growth whereas it is associated with its phosphorylated cytosolic target proteins in the stationary phase. Thus, the localization of PtkA is highly dynamic (Jers *et al.*, 2010).

The clustering of BY-kinases with genes required for extracellular polysaccharide matrix and/or capsule production is well established in a variety of bacteria such as *Escherichia coli*, *Staphylococcus aureus* and *Streptococcus pneumoniae* (Bechet *et al.*, 2010; Morona *et al.*, 2003; Soulat *et al.*, 2007; Wugeditsch *et al.*, 2001). Although the precise function of the BY-kinase in extracellular polysaccharide synthesis remains elusive, it is generally assumed that autophosphorylation of the BY-kinases is required for the export of polysaccharides (Bechet *et al.*, 2010; Whitfield, 2006).

Recently, we have shown that protein tyrosine phosphorylation is also implicated in biofilm formation in *Bacillus subtilis* (Kiley & Stanley-Wall, 2010). In the absence of the BY-kinase PtkA, the complex colonies are strongly wrinkled, but lack the rough outer region of the colony where usually the fruiting bodies are formed. This phenotype was attributed to the kinase activity of PtkA that is required for efficient sporulation under conditions of biofilm formation. In this study, we have investigated the potential role of the putative BY-kinase EpsB and its transmembrane modulator protein EpsA. Based on the similarity of EpsB and PtkA and their presumptive modulators, we have also investigated whether these proteins act in concert to control biofilm formation. Our results indicate that EpsB and PtkA fulfil distinct but additive functions in biofilm formation and that they require their cognate modulators for this.

## Methods

### 

#### Bacterial strains and growth conditions.

All *Bacillus subtilis* strains used in this work are derived from the laboratory wild-type strain 168 or the non-domesticated strain NCIB3610. Mutations were transferred to the NCIB3610 background using SPP1-mediated generalized transduction (Yasbin & Young, 1974). All strains are listed in Table 1. *E. coli* XL1-Blue (Stratagene) was used for plasmid constructions and transformation using standard techniques (Sambrook *et al.*, 1989).

**Table 1.** *Bacillus subtilis* strains used in this study

Strain	Genotype	Source/construction†‡
168	*trpC2*	Laboratory collection
8G5 *sinR* : : *tet*	*trpC2 tyr-1 his ade met rib ura nic sinR : : tet*	Oscar Kuipers, Groningen, the Netherlands
AM373	*sfp*^+^ *ermC epsC*^+^ *swrA*^+^ *degQ*^+^ *amyE* : : (*P-rapP phrP cat*) (*yvzG/yvyD spc)*	McLoon *et al*. (2011)
GP736	*trpC2* Δ*sinR : : tet*	8G5 *sinR* : : *tet* → 168
GP1517	*trpC2* Δ*epsA : : aphA3*	this work
GP1518	*trpC2* Δ*epsB : : aphA3*	this work
GP1519	*trpC2* Δ*epsAB : : aphA3*	this work
GP1526	*trpC2 epsA-3x FLAG spc*	pGP2127 → 168
GP1528	*trpC2 sfp*^+^ *ermC epsC^+^ swrA*^+^ *degQ*^+^ *amyE* : : (*P-rapP phrP cat)*(*yvzG*/*yvyD* Ω*spc*) Δ*epsAB* : : *aphA3*	GP1519 → AM373
GP1535	*trpC2 sfp*^+^ *ermC epsC^+^ swrA*^+^ *degQ*^+^ *amyE* : : (*P-rapP phrP cat)*(*yvzG*/*yvyD* Ω*spc*) Δ*epsB* : : *aphA3*	GP1518 → AM373
GP1540	*trpC2 sfp*^+^ *ermC epsC^+^ swrA*^+^ *degQ*^+^ *amyE* : : (*P-rapP phrP cat)*(*yvzG*/*yvyD* Ω*spc*) Δ*epsA* : : *aphA3*	GP1517 → AM373
GP1542	*trpC2 xkdE* : : P*_xyl_-epsB ermC*	pGP2129 → 168
GP1566	*trpC2* Δ*tkmA* : : *spc*	this work
GP1567	*trpC2* Δ*epsA* : : *aphA3* Δ*tkmA* : : *spc*	GP1566 → GP1517
GP1568	*trpC2* Δ*epsA* : : *aphA3* Δ*tkmA* : : *spc xkdE* : : *P_xyl_-epsA ermC*	pGP2147 → GP1567
GP1575	Δ*epsB* : : *aphA3*	GP1535 → NCIB3610*
GP1577	Δ*ptkA* Δ*epsB* : : *aphA3*	GP1535 → NRS2544*
GP1589	*trpC2 sinR : : tet epsA-3x FLAG spc*	GP736 → GP1526
GP1600	Δ*epsA* : : *aphA3*	GP1540 → NCIB3610*
GP1602	*tkmA* : : *spc*	GP1566 → NCIB3610*
GP1611	Δ*epsA* : : *aphA3 tkmA* : : *spc*	GP1566 → GP1600*
GP1622	Δ*sinR*-*tasA* : : *cat*	GP1672 → NCIB3610*
GP1623	Δ*epsB* : : *aphA3* Δ*sinR*-*tasA* : : *cat*	GP1672 → GP1575*
GP1624	Δ*ptkA* Δ*sinR*-*tasA* : : *cat*	GP1672 → NRS2544*
GP1625	Δ*epsB* : : *aphA3* Δ*ptkA* Δ*sinR*-*tasA* : : *cat*	GP1672 → GP1577*
GP1626	Δ*epsA* : : *aphA3* Δ*sinR*-*tasA* : : *cat*	GP1672 → GP1600*
GP1627	Δ*tkmA* : : *spc* Δ*sinR*-*tasA* : : *cat*	GP1672 → GP1602*
GP1628	Δ*epsA* : : *aphA3* Δ*tkmA* : : *spc* Δ*sinR*-*tasA* : : *cat*	GP1672 → GP1611*
GP1629	Δ*epsA-O* : : *tet* Δ*sinR*-*tasA* : : *cat*	GP1672 → NRS2450*
GP1634	Δ*epsB* : : *aphA3* Δ*ptkA xkdE* : : *P_xyl_-epsB ermC*	GP1542 → GP1577*
GP1636	Δ*epsA* : : *aphA3* Δ*tkmA* : : *spc xkdE : : P_xyl_-epsA ermC*	GP1568 → GP1611*
GP1637	Δ*epsAB* : : *aphA3*	GP1528 → NCIB3610*
GP1672	*trpC2* Δ*sinR*-*tasA* : : *cat*	this work
NRS2450	Δ*epsA-O* : : *tet*	Ostrowski *et al*. (2011)
NRS2499	*epsB* (D^81^A+D^83^A)	pNW329 → NCIB3610*
NRS2544	Δ*ptkA*	Kiley & Stanley-Wall (2010)

† Arrows indicate construction by transformation.

‡ Asterisks indicate construction by SPP1 phage transduction. BSGC represents the *Bacillus* genetic stock centre. Antibiotic resistance cassettes are: *aphA3*, kanamycin; *cat*, chloramphenicol; *tet*, tetracycline; *spc*, spectinomycin; *ermC*, erythromycin.

Luria–Bertani (LB) broth was used to grow *E. coli* and *Bacillus subtilis*. When required, media were supplemented with the following antibiotics – for *E. coli*: ampicillin (100 µg ml^−1^); and for *Bacillus subtilis*: spectinomycin (150 µg ml^−1^), kanamycin (10 µg ml^−1^), chloramphenicol (5 µg ml^−1^), erythromycin plus lincomycin (2 and 25 µg ml^−1^, respectively) and tetracycline (12 µg ml^−1^).

*Bacillus subtilis* was grown in C minimal medium supplemented with glucose, succinate, glutamate and auxotrophic requirements (at 50 mg l^−1^) (Commichau *et al.*, 2007). SP (sporulation) and MSgg plates were prepared by the addition of 17 g Bacto agar l^−1^ (Difco) to SP (8 g nutrient broth, 1 mM MgSO_4_ and 13 mM KCl l^−1^, supplemented after sterilization with 2.5 µM FeSO_4_, 500 µM CaCl_2_ and 10 µM MnCl_2_) or 15 g Bacto agar l^−1^ (Difco) to MSgg medium (Branda *et al.*, 2001).

#### Assays of complex colony and pellicle formation.

For colony architecture analysis, bacteria were precultured in LB to an OD_600_ of 0.6–0.8. Then, 10 µl of this cell suspension was spotted onto minimal MSgg medium (Branda *et al.*, 2001) containing 1.5 % agar and incubated at 22 °C for 3–5 days or at 37 °C for 48 h as indicated. To study the formation of pellicles, 8 µl of the above-mentioned preculture was used to inoculate 8 ml liquid MSgg medium and incubated at room temperature for 3–4 days.

#### DNA manipulation and transformation.

Plasmid DNA extraction was performed using standard procedures (Sambrook *et al.*, 1989). Restriction enzymes, T4 DNA ligase and DNA polymerases were used as recommended by the manufacturers. DNA fragments were purified from agarose gels using the QIAquick PCR purification kit (Qiagen). Phusion DNA polymerase was used for the PCR as recommended by the manufacturer. Primer sequences are detailed in Table S1 (available in the online Supplementary Material). DNA sequences were determined using the dideoxy chain-termination method (Sambrook *et al.*, 1989). All plasmid inserts derived from PCR products were verified by DNA sequencing. Standard procedures were used to transform *E. coli* (Sambrook *et al.*, 1989) and transformants were selected on LB plates containing ampicillin (100 µg ml^−1^) or kanamycin (50 µg ml^−1^).

Chromosomal DNA of *Bacillus subtilis* was isolated using the DNeasy Tissue kit (Qiagen) according to the supplier’s protocol. *Bacillus subtilis* was transformed with plasmid or chromosomal DNA according to the two-step protocol (Kunst & Rapoport, 1995). Transformants were selected on either LB or SP plates containing chloramphenicol (5 µg ml^−1^), kanamycin (10 µg ml^−1^), spectinomycin (150 µg ml^−1^), erythromycin plus lincomycin (2 and 25 µg ml^−1^, respectively) or tetracycline (12 µg ml^−1^).

#### Regulated expression of genes.

To allow the controlled expression of genes, we placed the gene behind a xylose-regulated promoter and integrated this cassette into the *xkdE* gene in the *Bacillus subtilis* chromosome. First, the origin of replication and the *bla* resistance gene were amplified from plasmid pUC19 (Sambrook *et al.*, 1989) using the primer pair KG50/KG51 (for primer sequences see Table S1). This fragment was ligated in a three-arm ligation to the flanking regions of the *Bacillus subtilis xkdE* gene (amplified with KG70/KG71 and KG72/KG73). The resulting plasmid was pGP883. Next, the *Bacillus subtilis xylA* promoter as well as the *ermC* erythromycin resistance gene from pDG647 (Guérout-Fleury *et al.*, 1995) were amplified (KG69/KG59 and KG48/KG49, respectively) and cloned between the *Bam*HI and *Sma*I sites of pGP883 in a three-arm ligation. With the oligonucleotide KG59, the translation initiation signals of the strongly expressed *gapA* gene were attached downstream of the *xylA* promoter. The resulting plasmid was pGP885. Then, the DNA region corresponding to the N-terminal fragment of the improved yellow fluorescent protein from plasmid pIYFP (Veening *et al.*, 2004) was amplified using the primer pair KG74/KG75. With KG74, a multiple cloning region encompassing *Xba*I, *Bam*HI, *Kpn*I and *Eco*RI sites was added. The fragment was cloned between the *Bam*HI and *Sal*I sites of pGP885 to give pGP886. A map of the vector pGP886 is available at http://subtiwiki.uni-goettingen.de/wiki/index.php/PGP886 (Michna *et al.*, 2014).

#### Construction of deletion and complementation strains.

Deletion of the *epsA*, *epsB* and *tkmA* genes as well as of the *epsAB* and *sinR–tasA* chromosomal regions was achieved by transformation with PCR products constructed using oligonucleotides (see Table S1) to amplify DNA fragments flanking the target genes and intervening antibiotic resistance cassettes (Guérout-Fleury *et al.*, 1995), as described by Wach (1996). To avoid polar effects on the expression of downstream genes of the *eps* and the *tkmA–ptkA–ptpZ–ugd* operons, we used resistance cassettes lacking a transcription terminator. Moreover, expression of the downstream *epsC* gene was verified in both the *epsA* and *epsB* mutants by quantitative real time reverse-transcription (qRT) RT-PCR. While the *epsC* gene was expressed as in the wild-type in the *epsA* mutant, the expression was slightly increased in the *epsB* mutant (data not shown), indicating that the downstream genes of the *eps* operon were expressed in both mutants.

To allow ectopic expression of *epsA* and *epsB*, we constructed the *Bacillus subtilis* strains GP1568 and GP1542, respectively. In these strains, the genes of interest are expressed in the *xkdE* locus under the control of the inducible xylose operon promoter P_Xyl_. For this purpose, we used the integrative complementation vector pGP886. The complementing plasmids were constructed as follows. The coding sequences of the *epsA* and *epsB* genes were amplified (for oligonucleotides, see Table S1), digested with *Xba*I and *Kpn*I and cloned into pGP886 linearized with the same enzymes. The resulting plasmids pGP2147 and pGP2129 were linearized with *Sca*I and *Not*I, respectively, and used to transform competent cells of *Bacillus subtilis* 168.

#### Construction of strains with chromosomal nucleotide substitutions.

Plasmid pNW323 was used for the construction of an in-frame, markerless point mutation of *epsB* at the ‘DxD’ motif. Primers NSW209 and NSW210 were used to amplify *epsB*, which was cloned into pCR2.1 (Invitrogen). Primers NSW213 and NSW214 were used to introduce point mutations into the plasmid. Following PCR, template DNA was digested using *Dpn*I. With the remaining DNA *E. coli* was transformed and colonies were selected based on ampicillin resistance. The resulting plasmid, pNW325, was sequenced to ensure that only the desired mutations were introduced. The region of DNA carrying *epsB* (D81A–D83A) was cloned into pMAD (Arnaud *et al.*, 2004) using the restriction sites engineered into primers NSW209 and NSW210. To introduce the mutations into NCIB3610, pNW329 was first transformed into *Bacillus subtilis* strain 168 and phage transduction was utilized to transfer the plasmid to *Bacillus subtilis* NCIB3610 (Kearns & Losick, 2003). NRS2499 was constructed by integration and curing of pNW329 in NCIB3610 (Arnaud *et al.*, 2004).

#### Construction of Δ*epsA*, Δ*epsB* and Δ*epsAB* mutant strains with an intact *epsC* gene.

In the laboratory strain, *Bacillus subtilis* 168, the *epsC* gene carries a point mutation and encodes an inactive protein (McLoon *et al.*, 2011). Any genetic transfer of constructed *epsA* and *epsB* alleles is likely to co-transfer this *epsC* mutation. Therefore, we constructed strains GP1540, GP1535 and GP1528 that carry a single deletion of *epsA* and *epsB* or a simultaneous deletion of *epsAB* and the *epsC* wild-type (*epsC*^+^) allele, respectively. For this, we transformed the *epsC*^+^ strain AM373 with chromosomal DNA of the *epsA* mutant GP1517, the *epsB* mutant GP1518 and the *epsAB* mutant GP1519. For the resulting Δ*epsA*, Δ*epsB* and *epsAB* transformants the chromosomal *epsC* allele of ten of the clones was analysed by sequencing, and in each case a single *epsC*^+^ transformant was re-isolated and termed GP1540, GP1535 and GP1528.

#### Precipitation and staining of exopolysaccharides (EPSs).

To analyse the formation of extracellular polysaccharides, precipitation and staining of polymers present in the culture supernatant were performed as described by Guttenplan *et al.* (2010). Briefly, we used the *sinR tasA* mutant to facilitate release of extracellular polysaccharides from the cell. The polysaccharides were precipitated using 75 % ethanol, separated from protein in the sample by SDS-PAGE and stained using the Stains-all dye (Applichem). In addition, polysaccharides in the supernatant were precipitated in 24-well plates by ethanol. For better visualization glycerol was added.

#### Real-time qRT-PCR.

For RNA isolation, cells were grown in CSE minimal medium containing 0.5 % (w/v) glucose (CSE-glucose) to an OD_600_ of 0.5–0.8 and harvested. Preparation of total RNA was carried out as described previously (Ludwig *et al.*, 2002). cDNAs were synthesized using the One-Step RT-PCR kit (Bio-Rad). qRT-PCR was carried out on an iCycler instrument (Bio-Rad) following the manufacturer’s recommended protocol by using the primers indicated in Table S1. The *rpsE* and *rpsJ* genes encoding constitutively expressed ribosomal proteins were used as internal controls. Data analysis and the calculation of expression ratios as fold changes were performed as described by Diethmaier *et al.* (2011). qRT-PCR experiments were performed in duplicate.

#### Spore quantification.

For the quantification of spores within biofilms we used the procedure described by Vlamakis *et al.* (2008). Briefly, complex colonies were collected, disrupted, sonicated and then plated in serial dilutions. To kill any surviving vegetative cells, aliquots were heated to 80 °C and plated again.

#### *In vivo* detection of protein–protein interactions.

To address the possible interaction between EpsA and EpsB, we used *Bacillus subtilis* GP1589/pGP2126, which encodes EpsA carrying a C-terminal FLAG-tag and EpsB with an N-terminal *Strep*-tag, to facilitate the purification and detection of the protein. Strain GP1526 was constructed as follows: The 3′ end of the *epsA* gene was amplified using the primer pair JG107/JG111, digested with *Bam*HI/*Pst*I and cloned into pGP1331 (Lehnik-Habrink *et al.*, 2010). The resulting plasmid pGP2127 was integrated into *Bacillus subtilis* 168, giving rise to strain GP1526. To ensure a high expression of the FLAG-tagged EpsA protein we deleted the *sinR* gene by transforming strain GP1526 with chromosomal DNA of strain GP736, resulting in strain GP1589. Plasmid pGP2126 was constructed by cloning the *epsB* gene between the *Bam*HI and *Sal*I sites of the expression vector pGP382 (Herzberg *et al.*, 2007).

The isolation of protein complexes from *Bacillus subtilis* cells was performed using SPINE technology (Herzberg *et al.*, 2007). Briefly, growing cultures of *Bacillus subtilis* were treated with formaldehyde (0.6 % w/v, 20 min) to facilitate cross-linking of interacting proteins (Herzberg *et al.*, 2007). The *Strep*-tagged proteins and their potential interaction partners were then purified from crude extracts using a Streptactin column (IBA) and desthiobiotin as the eluent. Interacting proteins were identified by Western blot analysis.

#### Western blotting.

For Western blot analysis, proteins were separated by 12 % SDS-PAGE and transferred onto PVDF membranes (Bio-Rad) by electroblotting. Rabbit anti-FLAG (Sigma-Aldrich; 1 : 10 000) polyclonal antibodies served as primary antibodies. The antibodies were visualized by using anti-rabbit immunoglobulin alkaline phosphatase secondary antibodies (Promega) and the CDP-Star detection system (Roche Diagnostics), as described previously (Commichau *et al.*, 2007).

#### Bacterial two-hybrid assay.

Primary protein–protein interactions were studied by bacterial two-hybrid (B2H) analysis. The B2H system is based on the interaction-mediated reconstruction of adenylate cyclase (CyaA) activity from *Bordetella pertussis* in *E. coli* (Karimova *et al.*, 1998). Briefly, proteins suspected to interact physically were fused with separated domains of the adenylate cyclase as described previously (Lehnik-Habrink *et al.*, 2010). DNA fragments corresponding to the *epsA* and *epsB* genes were obtained by PCR (for primers, see Table S1). The PCR products were digested with *Kpn*I and *Xba*I and cloned into the vectors of the two-hybrid system that had been linearized with the same enzymes. The resulting plasmids (see Table S2) were used for co-transformations of *E. coli* BTH101, and the protein–protein interactions were then analysed by plating the cells on LB plates containing ampicillin (100 µg ml^−1^), kanamycin (50 µg ml^−1^), X-Gal (80 µg ml^−1^) and IPTG (0.5 mM), respectively. The plates were incubated for a maximum of 48 h at 30 °C.

## Results

### *In vivo* interaction between EpsA and EpsB

**Fig. 1.**
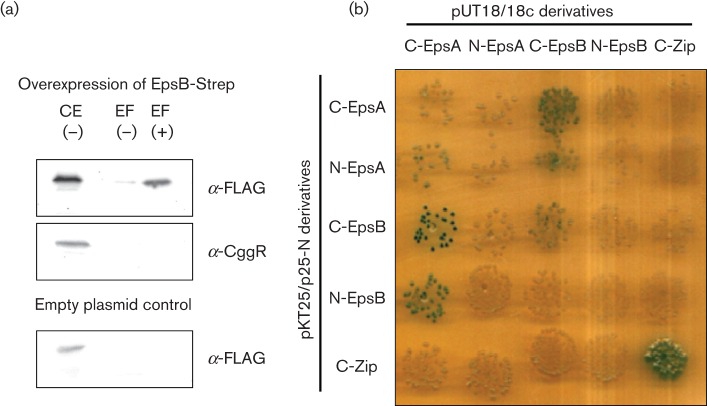
The tyrosine kinase EpsB and its cognate modulator protein EpsA interact physically. (a) EpsA co-purifies with EpsB. The EpsB-*Strep* fusion protein was expressed from plasmid pGP2126 under the control of a strong constitutive promoter in *Bacillus subtilis* GP1589 that expresses EpsA-FLAG under the control of its native promoter. To ensure high expression of EpsA-FLAG, the gene for the anti-activator SinR was deleted in this strain. Cells were grown in CSE-glucose medium until the late exponential growth phase. EpsB-*Strep* was purified in the absence (−) or presence (+) of the cross-linker formaldehyde. To detect the co-purified EpsA-FLAG protein, the elution fractions were heated to reverse the cross-linking and applied to a 12 % SDS polyacrylamide gel. After electrophoresis and blotting onto a PVDF membrane, EpsA was detected via its FLAG-tag. To test that the binding of EpsA to EpsB is not unspecific we tried to detect the CggR protein in the same elution fractions with a specific antibody. Additionally, we used an empty vector control to test unspecific binding of EpsA to the Streptactin column. CE, crude extract; EF, elution fraction. (b) EpsA and EpsB interact in the B2H system. The genes encoding EpsA and EpsB were cloned in the low-copy plasmids p25N and pKT25 and the high-copy plasmids pUT18 and pUT18c. These plasmids allow the expression of the genes of interest fused to the N or C terminus of the T18 or T25 domains of the *Bordetella pertussis* adenylate cyclase, respectively. The *E. coli* transformants harbouring both vectors were incubated for 48 h at 30 °C. Degradation of X-Gal and the resulting blue colour of the cells indicate interaction due to the presence of a functional adenylate cyclase.

The EpsA and EpsB proteins are similar to the TkmA transmembrane kinase modulator and the PtkA protein tyrosine kinase of *Bacillus subtilis*, respectively. It is well established that the modulator proteins stimulate the activity of their cognate protein kinases by protein–protein interaction. To address whether this is also the case for EpsA and EpsB, we analysed the potential interaction between these two proteins. For this, we used strain GP1589 carrying plasmid pGP2126. In this strain, EpsB is fused to an N-terminal *Strep*-tag and EpsA carries a C-terminal FLAG-tag. Additionally, the gene for the anti-activator (and master regulator of biofilm formation) SinR was deleted to ensure a high expression level of EpsA. If the two proteins interacted with each other, one would expect that the FLAG-tag epitope bound to EpsA is detectable in the elution fractions containing *Strep*-EpsB. As EpsA contains two transmembrane domains and is likely to be a membrane protein, the possible interaction between EpsA and EpsB was fixed using formaldehyde as cross-linker. *Strep*-EpsB with its bound interaction partners was purified by its binding to Streptactin columns. Both the cell extract and the elution fractions were analysed by SDS-PAGE and subjected to Western blot analysis. As shown in Fig. 1(a), EpsA-FLAG was present in the crude extract. Importantly, EpsA-FLAG co-eluted with EpsB in a protein preparation obtained with cross-linking. To ascertain the specificity of the binding of EpsA to EpsB, we tested whether CggR, a cytoplasmic transcription factor, was also co-purified with EpsB. As shown in Fig. 1(a), CggR was expressed under the tested conditions. However, CggR did not co-elute with EpsB. Additionally, we used an empty vector control to ensure that EpsA-FLAG does not bind to the Streptactin columns unspecifically. In this case we also could not detect EpsA-FLAG in the elution fractions. Therefore, the elution of EpsA is caused by a specific interaction with EpsB.

The results presented above demonstrate an interaction between the membrane protein EpsA and the putative protein kinase EpsB. However, they do not allow us to conclude whether this interaction is direct or indirect. To address this question, we studied the interaction using the B2H system. As shown in Fig. 1(b), EpsA and EpsB clearly interacted with each other in this heterologous *E. coli* system whereas neither of the two proteins exhibited an interaction with the control protein (leucine zipper of yeast Gcn4p). Thus, EpsA and EpsB are capable of interacting specifically and directly with each other.

### The role of tyrosine protein kinases in complex colony and pellicle formation

**Fig. 2.**
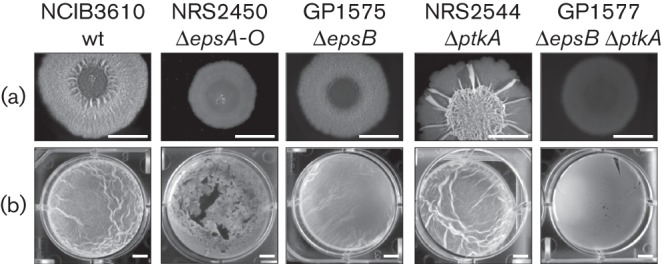
The deletion of tyrosine kinase genes in the wild-type strain NCIB3610 leads to less structured colonies and pellicles. (a) Complex colony formation. Cells were grown on MSgg agar plates solidified with 1.5 % agar for 1 day at 30 °C and for 1–2 days at room temperature prior to photography. Bars, 5 mm. (b) Pellicle formation. Cells were grown in MSgg medium for 2–3 days at room temperature prior to photography. Bars, 5 mm.

It is well established that BY-kinases are implicated in extracellular polysaccharide synthesis in many species (Grangeasse *et al.*, 2012). The location of the *epsA* and *epsB* genes in the *eps* operon encoding the functions for extracellular polysaccharide formation in *Bacillus subtilis* biofilms was highly suggestive of a role for these proteins in biofilm formation. To address the role of the putative BY-kinase EpsB in biofilm formation, we deleted the *epsB* gene in the undomesticated NCIB3610 wild-type strain. The resulting mutant was GP1575 (see Table 1). In agreement with previous reports (Branda *et al.*, 2001), the wild-type strain formed well-structured colonies (Fig. 2a) and thick and wrinkled pellicles (Fig. 2b). The isogenic *epsB* mutant GP1575 also formed structured colonies; however, the wrinkles resulting from EPS accumulation were completely lost (Fig. 2a). Similarly, the pellicle formed by the *epsB* mutant strain was less structured than observed for the wild-type (see Fig. 2b). The *epsB* deletion did not, however, have an impact as significant as the deletion of the entire *epsA-O* operon, thus suggesting that EPS biosynthesis was reduced but not completely lost in the *epsB* mutant. To rule out the possibility that the replacement of the *epsB* gene by an *aphA3* resistance cassette might have a polar effect on the expression of the downstream genes of the *eps* operon we used a resistance cassette that lacked a transcription terminator downstream of the *aphA3* gene. Moreover, we compared the expression of the downstream *epsC* gene in the wild-type strain 168 and the *epsB* mutant GP1518 by qRT-PCR. The *epsC* expression was not affected by the *epsB* deletion.

**Fig. 3.**
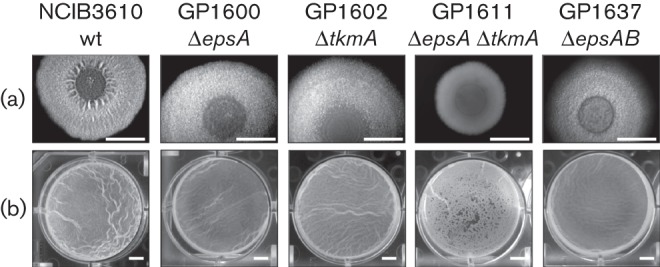
The deletion of tyrosine kinase modulator genes in the wild-type strain NCIB3610 leads to less structured colonies and pellicles. (a) Complex colony formation. Cells were grown on MSgg agar plates solidified with 1.5 % agar for 1 day at 30 °C and for 1–2 days at room temperature prior to photography. Bars, 5 mm. (b) Pellicle formation. Cells were grown in MSgg medium for 2–3 days at room temperature prior to photography. Bars, 5 mm.

To support our hypothesis that the transmembrane modulator EpsA is required for the function of the cognate EpsB protein, we deleted the *epsA* gene in the undomesticated NCIB3610 wild-type strain. The resulting mutant was GP1600 (see Table 1). As shown for the *epsB* mutant, the *epsA* mutant strain formed structured colonies but the wrinkles were lost (Fig. 3a). Also, the pellicle of the *epsA* mutant strain looked similar to pellicles formed by the *epsB* mutant (Fig. 3b). The observation that an *epsA* mutant has the same phenotype as an *epsB* mutant suggests that EpsA and EpsB act in one pathway of biofilm formation and supports the idea that the EpsA modulator is required for the function of the EpsB protein. To further study the idea that EpsA and EpsB act in one pathway of biofilm formation, the *epsAB* mutant GP1637 (see Table 1) was constructed and the biofilm phenotype was analysed. As expected, the *epsAB* double mutant showed the same phenotype as *epsA* and *epsB* single mutants, supporting the initial idea.

As EpsB and PtkA are the only BY-kinases in *Bacillus subtilis.* we addressed the question of whether both proteins have complementary function for biofilm formation. Therefore, we compared the effect of the inactivation of *epsB* with that of a *ptkA* mutation and studied the phenotype resulting from a loss of both BY-kinases. As observed previously (Kiley & Stanley-Wall, 2010), the *ptkA* mutant NRS2544 formed structured and strongly wrinkled colonies; however, these colonies lacked a rough outer region that is usually the area of sporulation and fruiting body formation (see Fig. 2a). The pellicles formed by the *ptkA* mutant were similar to those of the isogenic wild-type strain (see Fig. 2b). The most severe phenotype was observed for the *epsB ptkA* double mutant GP1577. As shown in Fig. 2(a), this strain was unable to form structured colonies, and was thus very similar to *epsA-O* or *ymdB* mutants (see Fig. 2; Diethmaier *et al.*, 2011).

Additionally, we performed a complementation assay with the *epsB ptkA* mutant strains that had a functional copy of the *epsB* gene under the control of a xylose-induced promoter in the non-essential *xkdE* locus (GP1634). Expression of the ectopic *epsB* gene upon addition of xylose to the biofilm medium restored the formation of a thick and structured pellicle (compare Figs S1 and S2). In conclusion, our data support the idea of a role for the BY-kinases in biofilm formation.

Interestingly, the simultaneous deletion of the genes coding for the two kinase modulators EpsA and TkmA in the NCIB3610 strain (GP1611) leads to the same phenotype as the deletion of both BY-kinases (Fig. 3). As shown for the *epsB ptkA* mutant, the ectopic expression of *epsA* in the *epsA tkmA* mutant (GP1636) also restored the formation of a thick and wrinkled pellicle (compare Figs S3 and S4). Again, this supports the idea that the two modulator proteins, EpsA and TkmA are required for the function of their cognate BY-kinases, EpsB and PtkA, respectively.

Together, the results reported above (i) clearly demonstrate the implication of EpsB and PtkA in biofilm formation and (ii) suggest distinct roles for the two enzymes in this process. As reported previously, PtkA seems to be required for biofilm-associated sporulation whereas EpsB is mainly responsible for EPS biosynthesis.

### Mutation of the active centre of EpsB does not impact sporulation

Biofilm formation by *Bacillus subtilis* involves cell fate differentiation and culminates in the formation of environmentally resistant spores (Lopez *et al.*, 2009; Vlamakis *et al.*, 2008). The formation of spores during biofilm formation is dependent on the synthesis of the extracellular matrix. The regulator Spo0A is required for both sporulation and biofilm formation (Lopez *et al.*, 2009). Spo0A is activated by phosphorylation upon detection of environmental stimuli that are perceived by sensor kinases. KinD links spore formation to extracellular matrix production (Aguilar *et al.*, 2010). To establish whether the BY-kinase EpsB has an impact on cell differentiation, we compared the level of sporulation of wild-type and mutant strains after 72 h of incubation under biofilm formation conditions (Vlamakis *et al.*, 2008). In contrast to the *epsA-O* deletion strain NRS2450 that was used as a control (mean±SEM 2.8±1 % sporulation), 95±0.1 % of the *epsB* mutant strain NRS2499 had sporulated; this level that was not statistically significantly different from the wild-type parental strain NCIB3610 (102±17 %). These findings support the idea that the impact of EpsB on biofilm formation is downstream from the checkpoint triggering sporulation during biofilm formation, which is controlled by KinD (Aguilar *et al.*, 2010).

### Tyrosine kinases influence extracellular polysaccharide production

**Fig. 4.**
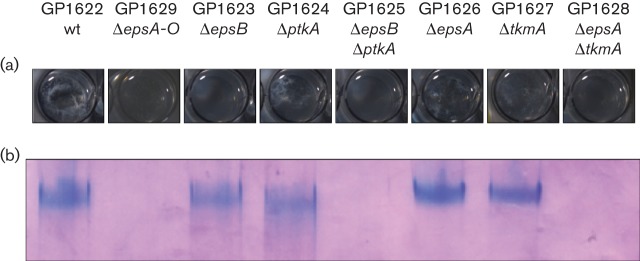
The deletion of tyrosine kinases affects EPS production. All strains contain a Δ*sinR*-*tasA* deletion to facilitate the release of EPSs into the culture medium. (a) Ethanol-precipitated supernatant from the indicated strains in the chambers of a 24-well plate. (b) Ethanol precipitates resolved in the stacker of an SDS-PAGE gel stained with Stains-all dye.

EPSs are a major component of the biofilm matrix (Branda *et al.*, 2001). The proteins for the synthesis and export of the EPS are encoded within the *epsA-O* operon. As shown in Fig. 2, deletion of the putative BY-kinase EpsB leads to a loss of the wrinkled colony and pellicle structure, which is usually considered as a loss of EPS production. The location of the *epsB* gene in the *epsA-O* operon supports this idea. To test our assumption that EpsB is involved in the production of EPSs, we used a strain with deletions of the *sinR* and *tasA* genes to enhance the production and release of EPSs from the cells. The cells were cultivated and the EPSs within the supernatant of the culture medium were precipitated by ethanol. Surprisingly, no major effect of the *epsB* deletion on the amount of EPSs was observed (Fig. 4). This suggests that either the putative kinase activity of EpsB is dispensable for EPS production or EpsB can be functionally replaced by the second BY-kinase, PtkA.

Therefore, the implication of the second BY-kinase PtkA in EPS production was addressed. In agreement with the wrinkled colony and pellicle structure of the *ptkA* mutant, similar amounts of EPSs as in the wild-type were observed. Thus, PtkA is not directly involved in the regulation of EPSs by the proteins encoded within the *epsA-O* operon. Interestingly, no EPS was detectable in the *epsB ptkA* double mutant. This observation is in perfect agreement with the complete lack of biofilm formation in the *epsB ptkA* double mutant and suggests that PtkA also affects EPS production, at least in the absence of EpsB.

Furthermore, we monitored EPS production of the *epsA* and *tkmA* modulator single mutants and the *epsA tkmA* double mutant. As shown for the respective BY-kinase mutants, the modulator single mutants are able to produce EPS, whereas the *epsA tkmA* mutant shows no EPS production. This supports the requirement of EpsB and PtkA for their modulator proteins.

## Discussion

We demonstrate here that the two protein tyrosine kinases of *Bacillus subtilis*, EpsB and PtkA, participate in biofilm formation. Inactivation of both kinases results in a complete loss of biofilm formation whereas the single mutants exhibit rather mild defects. Analysis of complex colony and pellicle formation also demonstrated that the two enzymes have distinct and complementary functions: PtkA is required to facilitate sporulation in the ‘fruiting body’-like structures in the outer region of the complex colonies (Kiley & Stanley-Wall, 2010). In contrast, EpsB is not involved in controlling sporulation. Instead, EpsB seems to be required for extracellular polysaccharide production. Together, both extracellular matrix production and sporulation seem to be important for the successful formation of a resistant biofilm.

Interestingly, we observed that EPS production is still active in the *epsB* mutant, whereas no EPS was formed in the *epsB ptkA* double mutant. Thus, it seems that PtkA can compensate for the loss of EpsB in the activation of extracellular polysaccharide synthesis. Such partial take-over of a function by a paralogous protein is not unprecedented: in *Bacillus subtilis* the HPr protein of the phosphotransferase system is involved in the phosphorylation and uptake of sugars. In addition, HPr phosphorylated at a specific serine residue acts as co-factor for the transcription factor CcpA, thus controlling carbon catabolite repression. In the absence of HPr, the paralogous protein Crh can partially replace HPr and bind to CcpA, although with significantly lower affinity. As observed here with EpsB and PtkA, this take-over of a function seems to be limited to situations in which the protein normally in charge of the function is absent whereas the paralogues normally have different functions.

It is well established that the BY-kinases require activation by transmembrane modules. These modules are either a domain of a larger kinase protein (as in the enzymes of Gram-negative bacteria) or separate proteins that are encoded by genes upstream of their cognate kinase genes (in *Firmicutes*) (Grangeasse *et al.*, 2012). In *Bacillus subtilis*, PtkA is activated by TkmA (Mijakovic *et al.*, 2003). Similar to the arrangement of the *tkmA* and *ptkA* genes, the *epsB* kinase-encoding gene is located downstream of the *epsA* gene coding for the modulator protein. Here, we have shown that the two proteins EpsA and EpsB do indeed physically interact *in vivo*, and that this interaction is direct (see Fig. 1). Moreover, our results demonstrate that the *epsA* mutant has phenotypes with respect to complex colony and pellicle formation identical to that of the *epsB* mutant. The genetic clustering, the physical interaction of the two proteins and the identity of mutant phenotypes indicate that EpsA is required for the presumptive protein kinase activity of EpsB.

For several bacterial tyrosine kinases it has been demonstrated that they regulate the activity, the localization or the interaction properties of proteins by the phosphorylation of their targets. This suggests that EpsB may also act by directly phosphorylating other proteins. This prompted us to perform an intensive search for phosphorylation targets of the BY-kinase EpsB; unfortunately, no phosphorylation targets could be identified.

To study the role of EpsA and EpsB in more detail, we will attempt to identify interaction partners of the two proteins and will analyse potential EpsB-dependent phosphorylation of these proteins.
